# Does Peer Reviewing for COVID-19-Related Papers Still Work?

**DOI:** 10.3389/frma.2020.571886

**Published:** 2020-10-08

**Authors:** Octavio Orellana-Serradell, Magda C. Díaz, María Fernanda González, Myriam Gutiérrez, Daniela Herrera, Daniela Jara, Diego Maureira, Jenny L. Ruiz-Fuentes, Sofía Sanhueza, Lisette Leyton

**Affiliations:** ^1^ Millennium Nucleus of Ion Channel-Associated Diseases (MiNICAD), Instituto de Ciencias Biomédicas, Facultad de Medicina, Universidad de Chile, Santiago, Chile; ^2^ Laboratorio de Mecanotransducción en La Fisiopatología Cardiaca, Programa de Fisiología y Biofísica, Instituto de Ciencias Biomédicas, Facultad de Medicina, Universidad de Chile, Santiago, Chile; ^3^ Grupo de Investigación en Ciencias Básicas y Clínicas de La Salud, Pontificia Universidad Javeriana de Cali, Cali, Colombia; ^4^ Cellular Communication Laboratory, Program of Cellular & Molecular Biology, Instituto de Ciencias Biomédicas, Facultad de Medicina, Universidad de Chile, Santiago, Chile; ^5^ Center for Studies of Exercise, Metabolism and Cancer (CEMC), Instituto de Ciencias Biomédicas, Facultad de Medicina, Universidad de Chile, Santiago, Chile; ^6^ Advanced Center for Chronic Diseases (ACCDiS), Instituto de Ciencias Biomédicas, Facultad de Medicina, Universidad de Chile, Santiago, Chile; ^7^ Programa de Doctorado en Ciencias Biomédicas, Escuela de Postgrado, Facultad de Medicina, Universidad de Chile, Santiago, Chile; ^8^ Laboratory of Cellular and Molecular Biology, Institute for Research in Dental Sciences, Faculty of Dentistry, Universidad de Chile, Santiago, Chile; ^9^ Cell Biology Laboratory, Program of Cellular & Molecular Biology, Instituto de Ciencias Biomédicas, Facultad de Medicina, Universidad de Chile, Santiago, Chile; ^10^ Laboratory of Molecular Cardiovascular Pathophysiology, Programa de Fisiología y Biofísica, Instituto de Ciencias Biomédicas, Facultad de Medicina, Universidad de Chile, Santiago, Chile; ^11^ Laboratory of Obesity and Metabolism in Geriatrics and Adults, Institute of Nutrition and Food Technology (INTA), Universidad de Chile, Santiago, Chile

**Keywords:** coronavirus, severe acute respiratory syndrome, SARS-CoV-2, infectious disease, pandemic, scientific rigor, research funds

## Introduction

In this article, we aimed to analyze whether coronavirus disease 2019 (COVID-19)-associated articles were being subjected to the same standards of peer-review as non-COVID-19 articles. In order to do this, we taught eight PhD students manuscript reviewing skills and analyzed eight papers published in valued journals, five of them on COVID-19. Each selected publication was reviewed by at least two graduate students from a Scientific English class and two scientists in charge of the course at the Faculty of Medicine, Universidad de Chile. Several shortcomings were identified in the revised studies, particularly on those related to COVID-19, which led us to conclude that the emergency imposed by the COVID-19 has endangered the quality of the accepted studies.

The COVID-19 pandemic caused by the novel Severe Acute Respiratory Syndrome Coronavirus 2 (SARS-CoV-2) has rapidly spread throughout the world. This virus is killing many people, and taking a massive physical, as well as mental toll, on the lives of all those that have been infected (Yi et al., 2020). The COVID-19 pandemic has severely depressed every country’s economy because governments have been obliged to apply quarantine measures to control the disease. Thus, the impact has not only been on people’s health, but also on their lifestyle and economic situation (Nicola et al., 2020). For these same reasons, many people, including politicians and leaders from different countries, have turned to the scientific community for answers regarding actions that need to be taken to control and treat the disease. Nevertheless, many scientific studies published these days concerning the COVID-19 virus, even those reported by important journals, fall short on experimental evidence to support their conclusions. It is clear that great pressure exists to rapidly know more about this virus and how to stop the pandemic. Notwithstanding, we believe that this is leading editors and reviewers to accept manuscripts that would have never been considered for publication under different circumstances. It is not bad science, it is just not the complete story; the story that the good, high impact journals would normally ask for, when peer reviewing manuscripts for publication.

## Article Analysis

In a Scientific English course for postgraduate students, we taught them how to review a manuscript and gave them several scientific papers on COVID-19 published in prestigious journals. Their task was to elaborate a critique, according to the instructions given by the professors. Additionally, non-COVID-19 articles (but of related subtopics) from similar journals were reviewed by the students as controls. All papers were evaluated by at least two students and reviewed by the two scientists in charge of the course.

Considering that: i) journals have received a wealth of manuscripts on COVID-19 and therefore, accelerated the publication reviewing process to allow faster publication, and dissemination of information (pandemic publishing) (Kwon, 2020); ii) the worldwide daily confirmed peak of COVID-19 deaths was at the middle of April 2020 (https://ourworldindata.org/grapher/daily-covid-deaths-region), and started rising in December 2019; and iii) data published in a middle-to-high impact journal (IF > 9; 2019) can cause more damage to the public because it is easier to consider the information as reliable and valid (Kwon, 2020), the papers were selected based on the following criteria: i) paper main topic was on COVID-19; ii) publication date was between January–June 2020; iii) articles were mainly brief reports; only one research article was selected; iv) papers were published by journals with an IF > 9.4 (2019). Additionally, as controls, we selected three papers meeting the same criteria, except they did not cover COVID-19.

To perform the critique, we used the criteria described under the sub-item “Critique” in Table 1. These criteria included: structure of the paper, data collection, appropriate methods and controls to gather the evidence, analysis and interpretation of evidence lead the reader to similar conclusions than the authors.

**TABLE 1:** Criteria used to evaluate the work performed by the students. Peer reviewing was performed as suggested by published literature (Benos et al., 2003; McPeek et al., 2009; Lippi, 2018).

**Comprehension and articulation of the summary**
Objective and accurate summary of author’s main points (Introduction)
Summary includes the author’s main points, including evidence provided to support his/her arguments
**Critique**
Reference to the structure of the article is provided (logical, well supported arguments)
Evaluation and critical judgment of the data collection, techniques are provided
Evaluation and critical judgment of how the data were analyzed are provided
Evaluation and critical judgment of how the data were interpreted by the authors are provided
Evaluation and critical judgment of how successful the authors were at making their point
Arguments to support your agreement or disagreement are provided
A general evaluation of the article is provided at the end
Format and style
Arguments are backed-up with appropriate references
Citations are complete (author, Journal, etc.)
Correct grammar, spelling, and punctuation
Writing is clear, logical and easy to follow
Use of paragraphing, devices to join sentences and ideas are appropriate

A paper written by Emmie de Wit and colleagues and published by *PNAS* in February 2020 (de Wit et al., 2020) was reviewed by all students. In this paper, the authors reported that prophylactic treatment with the antiviral drug Remdesivir prevented clinical manifestations in the lungs of *Rhesus macaques* infected with MERS-CoV, and provided a clear clinical benefit when the drug was administered post infection. They suggest that Remdesivir could be useful in the treatment of other coronaviruses such as SARS-CoV-2, the virus responsible for COVID-19. Although the article is well backed up, there were a number of important concerns. First, the number of animals in each group of the study was very small (six), which led to a considerable variation in the results observed, making the reported therapeutic effect of Remdesivir questionable. In addition, although the authors had two different types of vehicle control groups (three animals/group), the results were treated as if these two groups were the same. Furthermore, the study lacked a control group without viral inoculation. Additionally, only male animals were used, despite the evidence indicating that adverse effects may vary between male and female animals (Klein, 2012). Finally, important clinical details about the macaques, such as their age—which is known to influence MERS-CoV infection outcome (Garbati et al., 2016)—weight, physical activity, presence of chronic diseases, etc., should have been indicated. Yet another limitation of this publication was the absence of toxicity assays. Measurements of renal clearance, liver or renal damage, and determinations of Remdesivir side effects, such as nausea, should have been included. Similar concerns were raised in the rest of the articles analyzed.

In another report by Giamarellos-Bourboulis and colleagues published in *Cell Host & Microbe* in April 2020 (Giamarellos-Bourboulis et al., 2020), the authors describe a unique signature of the immune response, different from that induced by bacterial community-acquired pneumonia sepsis or H1N1 influenza, which precedes severe respiratory failure in COVID-19 patients. The common critique to this study was the lack of healthy controls in some of the experiments, which appeared in only a few of the comparisons performed throughout the article. Healthy controls should have been included in all the analyses, and their clinical data, provided. Additionally, some of the conclusions were drawn using data that appears as non-significant in the corresponding graphs. For example, Figure 2E shows no statistically significant differences between B lymphocyte counts, when comparing immune dysregulated patient samples and intermediate state patients or healthy controls. Therefore, the authors should not have drawn the conclusion of lymphopenia as a characteristic of COVID-19 patients with immune dysregulation. Moreover, many of the figures have high data dispersion, and some of them even show outliers. Statistical outcomes obtained using data sets that include outliers can often be misleading and compromise the generalizability of the research findings (Salgado et al., 2016).

Another example is the study by Hoffmann and colleagues published in *Molecular Cell* in April 2020 (Hoffmann et al., 2020). The authors demonstrate the importance of a multibasic site in the SARS-CoV-2 spike protein, for proteolytic cleavage. They identify the endo protease Furin as a potential target for therapeutic intervention, since this protease cleaves the S protein, which is a key step for viral entry into lung cells. However, the quantitative densitometric analyses of the immunoblots showing cleavage comparison were not provided, making it hard to extrapolate their conclusions to the actual COVID-19 condition. Furthermore, this study lacked physiologically relevant models, such as a primary lung cell line or an *in vivo* system, in order to test the different mutations of the multibasic site. The use of a more appropriate model would have permitted a rigorous evaluation of how these mutations affect viral infection. In addition, an *in vivo* approach would also have been useful to test the effects of the Furin inhibitor, a shortcoming the authors themselves acknowledge in the discussion section, considering that the drug may exert toxic effects. Thus, suggesting Furin as a COVID-19 therapeutic target seemed rather premature.

The brief communication published in *Nature Medicine* in April 2020 by Leung et al. (2020) discusses the efficacy of face masks in preventing transmission of three different viruses, including COVID-19. A major concern here was that the authors did not clearly describe the masks used in the experiments, particularly, in terms of the submicron-sized filter or the mask certification. This information is highly relevant because differences have been reported between different face masks and their ability to filter aerosols (Oberg and Brosseau, 2008). Moreover, differences between aerosols and droplet transmission were not discussed and although the authors concluded that for all studied viruses shedding is higher in nasal swabs than in throat swabs, they did not provide a statistical analysis of these results. Another important problem was the small size of the population analyzed for coronavirus (only 17 patients) and the fact that this sample included patients with chronic medical conditions (five patients) and one smoker. These conditions may cause changes in the respiratory rate and other symptoms that might not be a direct consequence of viral infection but of the underlying condition (Martin et al., 2016; Britto et al., 2017). As an outcome, the number of viral copies in exhaled breath could be altered and might not reflect the real values of most patients. Furthermore, swab samples were taken from all 17 patients; however, in the droplet vs. aerosol experiments, the number of patients was reduced to 10 (without mask) and 9 (with mask), respectively. This limited sample size is insufficient to draw significant conclusions.

The last report on COVID-19 reviewed was the one published in *Science* by Rockx et al. (2020) in April 2020. Here, the authors studied the pathogenesis of infection produced by SARS-CoV-2 and compared it with that of SARS-CoV and MERS-CoV, using a non-human primate infection model. A common critique here was that the information provided about the studied subjects was incomplete, considering that the main goal of the article was to describe an appropriate animal model for COVID-19 trials. No information concerning the macaques, such as physical status or health condition was provided, although these are parameters that can affect the severity of respiratory diseases. Moreover, the authors did not provide information concerning the exact age of macaques in both MERS and SARS groups, nor did they mention specific details about the inoculation doses used in the experiments. Moreover, the data showed high variability and no statistical analyses were provided.

The first article reviewed as a control was published in *Cell Host & Microbe* by Di Luccia and colleagues, on June 2020 (Di Luccia et al., 2020). The authors studied the effect that undernutrition and microbiota can have on the immune response to oral vaccination in a gnotobiotic mouse model. In this article, we observed that sample collection and clinical data from the donors, the methodology regarding the mouse model, controls and statistics were well detailed. Nevertheless, a common critique found was that the study included only fecal samples from one child donor for each group of study (supplement-responsive and supplement-hyporesponsive).

Another article we used as a control was published in *PNAS* by Wang and colleagues, on June 2020 (Wang et al., 2020). Here, the authors compared the capacity to lower the viral load of wild-type anti–HIV-1 immunoglobulin G1, using an Fc Null variant of the same antibody in both a humanized mouse model and in *R. macaques*. The article was well written, the methodology was explained with enough detail, and the controls and sample sizes used were appropriate. The only critique made was that the confirmation of the results using different antibodies were performed using distinct virus strains.

Our last article reviewed as a control was published in *Nature Medicine*, in March 2020. Here, Colby and colleagues provide evidence for the safety, immunogenicity, and viral rebound dynamics of a heterologous Ad26, MVA vaccine regimen in antiretroviral therapy-suppressed HIV patients (Colby et al., 2020). The major criticism found here was that the authors did not perform power calculations to obtain an optimal sample size, which they mention was due to the number of available subjects. Furthermore, they used the Wilcoxon test (which compares two related samples) for statistical significance even though their graphs show the comparison of multiple populations. Additionally, they only studied Asian males, which confers, gender and ethnic biases to their results.

Therefore, major criticisms that were common to most of the reports on COVID-19 were: i) high variability of the results, or no statistical analysis provided, or results with non-significant differences; ii) lack of appropriate controls; iii) models were not described in detail, incomplete patient information, insufficient information about the experimental design, exact age or lack of information related to doses inoculated, etc.; iv) small sample size; v) no indication of toxicity assays; vi) insufficient evidence provided to claim clinical relevance; vii) a more physiologically relevant model would have been necessary to draw the conclusions. All of the aforementioned elements are crucial requisites that should not be bypassed in the peer reviewing process. Nevertheless, we did notice a greater number of these issues in COVID-19 related papers than in control papers. Additionally, the students also noted that non-COVID-19 articles were more prone to discussing the limitations of the studies and less prone to overselling their results in comparison to COVID-19 related ones. Importantly, a more extended reviewing process was evident for the control articles (>50 days) when compared to the COVID-19 papers (mean = 31 days) (Table 2).

**TABLE 2:** Summary of selected articles, indicating the journal and its impact factor, along with the number of days to final acceptance, and the article type, i.e., short (green) vs. long (orange) articles.

Paper on COVID-19?	Journal (IF 2019)	Dates of reception/acceptance	Number of days	Article type
Yes	*Molecular Cell* (15, 5)	Received March 15, 2020; accepted April 17, 2020	33	Short article
Yes	*Nature Medicine* (36)	Received February 3, 2020; accepted February 20, 2020	17	Brief communication
Yes	*Cell Host & Microbe* (15, 9)	Received March 19, 2020; accepted April 9, 2020	21	Report
Yes	*Science* (40)	Received March 15, 2020; accepted April 15, 2020	31	Report
Yes	*PNAS* (9, 41)	Received December 16, 2019; accepted February 7, 2020	56	Research article
No	*Nature Medicine* (36)	Received May 6, 2019; accepted January 24, 2020	262	Brief communication
No	*PNAS* (9, 41)	Received April 27, 2020; accepted June 17, 2020	51	Research article
No	*Cell Host & Microbe* (15, 9)	Received February 14, 2020; accepted April 8, 2020	54	Short article

## Discussion

Scientific rigor is a must no matter the circumstances. Thus, researchers and scientific journals should not take advantage of the pandemic contingency to publish papers that do not present enough evidence to support the conclusions claimed by the authors. Particularly, in these times when people turn to scientists in search of answers to calm their fears and concerns. The peer review process should assist the scientific community in “assuring the quality of research before it is published and before it can be examined and used by a wider audience” (Cargill and O’Connor, 2013). Therefore, peer reviewing should be carried out thoroughly and meticulously to guarantee that carefully conducted scientific studies are being published in these emergency times.

In addition, because the situation is critical and it will inevitably affect the economy worldwide, significant financial cuts are foreseen in every field and discipline, and science is not an exception. However, this is a typical catch-twenty-two situation. Science, research, and experimentation is needed to learn about all these microbes, viruses, and other microorganisms that can cause severe damage to human health, yet funds are being cut in order to provide money for other more immediate needs. What all the relevant players need to learn from this experience, is that science should always be an action rather than a reaction, which is what we are now learning from these articles that have been peer reviewed using less than rigorous criteria. Likewise, while fear of running out of funds and the urgent need for a treatment for this deadly disease may be pressuring from all angles to publish at any cost, ethics and rigor are core scientific values that need to be met to draw meaningful conclusions.

Interestingly, when the students were asked to review additional non-COVID-19 articles as controls for this Opinion, they rapidly noticed the apparent differences in the details between COVID-19 and non-COVID-19 articles with respect to the methodology used and other significant issues analyzed during this study (see Table 1). Thus, the question as to whether COVID-19 articles, given the current pandemic, are being reviewed with a less critical eye is supported here by the comparison performed with the non-COVID-19 article reviews. Until now, other authors have also remarked on this issue (Bagdasarian et al., 2020; Kwon, 2020). However, it is noteworthy to point out that peer reviewing is a complex task that involves human judgment and interpretation of someone else’s experimental design and findings, and as such, it is not free of error (e.g, see http://retractionwatch.com/).

## Author Contributions

The authors confirm to have contributed to this work and have all approved it for publication.

## Funding

LL was funded by the Agencia Nacional de Investigación y Desarrollo (ANID) grant (FCYT1200836), OO was funded by the Millennium Nucleus of Ion Channel-Associated Diseases (MiNICAD), and the PhD students by ANID fellowships [Beca Doctorado Nacional 21190214 (DJ); 21170292 (MFG); 21201941 (DM); 21191668 (DH); 21190725 (JR); 21191341 (MD), and tuition fee/stipend grant for Doctoral Studies 2020, from the Faculty of Medicine, Universidad de Chile (MG, SS)].

## Conflict of Interest

The authors declare that the research was conducted in the absence of any commercial or financial relationships that could be construed as a potential conflict of interest.

## References

[B1] Bagdasarian N. Cross G. B. Fisher D. (2020). Rapid publications risk the integrity of science in the era of COVID-19. BMC Med. 18, 192. 10.1186/s12916-020-01650-6 32586327 PMC7315694

[B2] Benos D. J. Kirk K. L. Hall J. E. (2003). How to review a paper. Adv. Physiol. Educ. 27, 47–52. 10.1152/advan.00057.2002 12760840

[B3] Britto C. J. Brady V. Lee S. Dela Cruz C. S. (2017). Respiratory viral infections in chronic lung diseases. Clin. Chest Med. 38, 87–96. 10.1016/j.ccm.2016.11.014 28159164 PMC5679206

[B4] Cargill M. O’Connor P. (2013). Writing scientific research articles: strategy and steps (Google eBook). 2nd Edn. Hoboken, NJ, USA: Blackwell Publishing Ltd.

[B5] Colby D. J. Sarnecki M. Barouch D. H. Tipsuk S. Stieh D. J. Kroon E. (2020). Safety and immunogenicity of Ad26 and MVA vaccines in acutely treated HIV and effect on viral rebound after antiretroviral therapy interruption. Nat. Med. 26, 498–501. 10.1038/s41591-020-0774-y 32235883

[B6] de Wit E. Feldmann F. Cronin J. Jordan R. Okumura A. Thomas T. (2020). Prophylactic and therapeutic remdesivir (GS-5734) treatment in the rhesus macaque model of MERS-CoV infection. Proc. Natl. Acad. Sci. U.S.A. 117, 6771–6776. 10.1073/pnas.1922083117 32054787 PMC7104368

[B7] Di Luccia B. Ahern P. P. Griffin N. W. Cheng J. Guruge J. L. Byrne A. E. (2020). Combined prebiotic and microbial intervention improves oral cholera vaccination responses in a mouse model of childhood undernutrition. Cell Host Microbe. 27, 899–908. 10.1016/j.chom.2020.04.008 32348782 PMC7292785

[B8] Garbati M. A. Fagbo S. F. Fang V. J. Skakni L. Joseph M. Wani T. A. (2016). A comparative study of clinical presentation and risk factors for adverse outcome in patients hospitalised with acute respiratory disease due to MERS coronavirus or other causes. PLoS One. 11, e0165978. 10.1371/journal.pone.0165978 27812197 PMC5094725

[B9] Giamarellos-Bourboulis E. J. Netea M. G. Rovina N. Akinosoglou K. Antoniadou A. Antonakos N. (2020). Complex immune dysregulation in COVID-19 patients with severe respiratory failure. Cell Host Microbe. 27, 992–1000. 10.1016/j.chom.2020.04.009 32320677 PMC7172841

[B10] Hoffmann M. Kleine-Weber H. Pöhlmann S. (2020). A multibasic cleavage site in the spike protein of SARS-CoV-2 is essential for infection of human lung cells. Mol. Cell. 78, 779–784. 10.1016/j.molcel.2020.04.022 32362314 PMC7194065

[B11] Klein S. L. (2012). Sex influences immune responses to viruses, and efficacy of prophylaxis and treatments for viral diseases. Bioessays 34, 1050–1059. 10.1002/bies.201200099 23012250 PMC4120666

[B12] Kwon D. (2020). How swamped preprint servers are blocking bad coronavirus research. Nature 581, 130–131. 10.1038/d41586-020-01394-6 32382120

[B13] Leung N. H. L. Chu D. K. W. Shiu E. Y. C. Chan K.-H. McDevitt J. J. Hau B. J. P. (2020). Respiratory virus shedding in exhaled breath and efficacy of face masks. Nat. Med. 26, 676–680. 10.1038/s41591-020-0843-2 32371934 PMC8238571

[B14] Lippi G. (2018). How do I peer-review a scientific article?—a personal perspective. 10.21037/atm.2017.12.15 PMC5879526 29610756

[B15] Martin E. M. Clapp P. W. Rebuli M. E. Pawlak E. A. Glista-Baker E. Benowitz N. L. (2016). E-cigarette use results in suppression of immune and inflammatory-response genes in nasal epithelial cells similar to cigarette smoke. Am. J. Physiol. Lung Cell Mol. Physiol. 311, L135–L144. 10.1152/ajplung.00170.2016 27288488 PMC4967187

[B16] McPeek M. A. DeAngelis D. L. Shaw R. G. Moore A. J. Rausher M. D. Strong D. R. (2009). The golden rule of reviewing. Am. Nat. 173, E155–E158. 10.1086/598847

[B17] Nicola M. Alsafi Z. Sohrabi C. Kerwan A. Al-Jabir A. Iosifidis C. (2020). The socio-economic implications of the coronavirus pandemic (COVID-19): a review. Int. J. Surg. 78, 185–193. 10.1016/j.ijsu.2020.04.018 32305533 PMC7162753

[B18] Oberg T. Brosseau L. M. (2008). Surgical mask filter and fit performance. Am. J. Infect. Contr. 36, 276–282. 10.1016/j.ajic.2007.07.008 PMC7115281 18455048

[B19] Rockx B. Kuiken T. Herfst S. Bestebroer T. Lamers M. M. Oude Munnink B. B. (2020). Comparative pathogenesis of COVID-19, MERS, and SARS in a nonhuman primate model. Science 368, 1012–1015. 10.1126/science.abb7314 32303590 PMC7164679

[B20] Salgado C. M. Azevedo C. Proença H. Vieira S. M. (2016). “Noise versus outliers,” in Secondary analysis of electronic health records (Cham, Switzerland: Springer International Publishing), 163–183. 10.1007/978-3-319-43742-2_14

[B21] Wang P. Gajjar M. R. Yu J. Padte N. N. Gettie A. Blanchard J. L. (2020). Quantifying the contribution of Fc-mediated effector functions to the antiviral activity of anti-HIV-1 IgG1 antibodies *in vivo* . Proc. Natl. Acad. Sci. U.S.A. 117, 18002. 10.1073/pnas.2008190117 32665438 PMC7395461

[B22] Yi Y. Lagniton P. N. P. Ye S. Li E. Xu R.-H. (2020). COVID-19: what has been learned and to be learned about the novel coronavirus disease. Int. J. Biol. Sci. 16, 1753–1766. 10.7150/ijbs.45134 32226295 PMC7098028

